# Detection and position evaluation of chest percutaneous drainage catheter on chest radiographs using deep learning

**DOI:** 10.1371/journal.pone.0305859

**Published:** 2024-08-12

**Authors:** Duk Ju Kim, In Chul Nam, Doo Ri Kim, Jeong Jae Kim, Im-kyung Hwang, Jeong Sub Lee, Sung Eun Park, Hyeonwoo Kim

**Affiliations:** 1 Department of Radiology, Jeju National University School of Medicine, Jeju Natuional University Hospital, Jeju, Republic of Korea; 2 Department of Radiology, Gyeongsang National University School of Medicine and Gyeongsang National University Changwon Hospital, Changwon, Republic of Korea; 3 Upstage AI, Yongin-si, Gyeonggi-do, Republic of Korea; Phramongkutklao College of Medicine, THAILAND

## Abstract

**Purpose:**

This study aimed to develop an algorithm for the automatic detecting chest percutaneous catheter drainage (PCD) and evaluating catheter positions on chest radiographs using deep learning.

**Methods:**

This retrospective study included 1,217 chest radiographs (proper positioned: 937; malpositioned: 280) from a total of 960 patients underwent chest PCD from October 2017 to February 2023. The tip location of the chest PCD was annotated using bounding boxes and classified as proper positioned and malpositioned. The radiographs were randomly allocated into the training, validation sets (total: 1,094 radiographs; proper positioned: 853 radiographs; malpositioned: 241 radiographs), and test datasets (total: 123 radiographs; proper positioned: 84 radiographs; malpositioned: 39 radiographs). The selected AI model was used to detect the catheter tip of chest PCD and evaluate the catheter’s position using the test dataset to distinguish between properly positioned and malpositioned cases. Its performance in detecting the catheter and assessing its position on chest radiographs was evaluated by per radiographs and per instances. The association between the position and function of the catheter during chest PCD was evaluated.

**Results:**

In per chest radiographs, the selected model’s accuracy was 0.88. The sensitivity and specificity were 0.86 and 0.92, respectively. In per instance, the selected model’s the mean Average Precision 50 (mAP50) was 0.86. The precision and recall were 0.90 and 0.79 respectively. Regarding the association between the position and function of the catheter during chest PCD, its sensitivity and specificity were 0.93 and 0.95, respectively.

**Conclusion:**

The artificial intelligence model for the automatic detection and evaluation of catheter position during chest PCD on chest radiographs demonstrated acceptable diagnostic performance and could assist radiologists and clinicians in the early detection of catheter malposition and malfunction during chest percutaneous catheter drainage.

## Introduction

Various types of intravascular tubes, such as peripherally inserted central catheters (PICC) or central venous catheters (CVC); airway tubes, such as endotracheal tubes (ETT); nasogastric tubes, such as Levin tubes; and drainage tubes, such as those used in percutaneous catheter drainage (PCD), are used in patients who are hospitalized, especially those in the intensive care unit [1–8]. As most of these tubes must be placed in an ideal position, assessing their current position is crucial. Computed-tomography (CT) is the plausible and practical methods for evaluating the position and pathway of PICC [9]. However, CT is not suitable due to its high cost and radiation exposure in clinical practice setting. Therefore, radiologists routinely assess tube position using radiographs owing to their widespread availability and cost-effectiveness. However, the tube position may not be assessed at an appropriate time if the hospital is understaffed or if the staff is occupied with other tasks. Several recent studies have used artificial intelligence (AI) [10–15] to assess the position of feeding tubes [11], ETT [14], PICC and CVC [10] on radiographs. However, no previous study has used automated methods to assess the position of the catheter during chest PCD on radiographs. Chest PCD is usually performed under fluoroscopic guidance to drain pleural effusion, empyema, hemothorax, pneumothorax, or lung abscess to relieve symptoms such as dyspnea or fever and to treat disease. Evaluating the position of the chest PCD is crucial, as malposition of the catheter increases the risk of malfunction. This could lead to worsening of the symptoms and the requirement for re-intervention. An automated solution, leveraging AI’s deep learning technology to evaluate the position of chest PCD on chest radiographs without involvement of radiologists or clinical physicians, can be used for the early identification of malpositioning to assist radiologists and clinical physicians. Therefore, this study aimed to develop an algorithm for the automatic detection and evaluation of the position of the catheter during chest PCD on chest radiographs using deep learning.

## Materials and methods

### Study design

**Fig 1:**
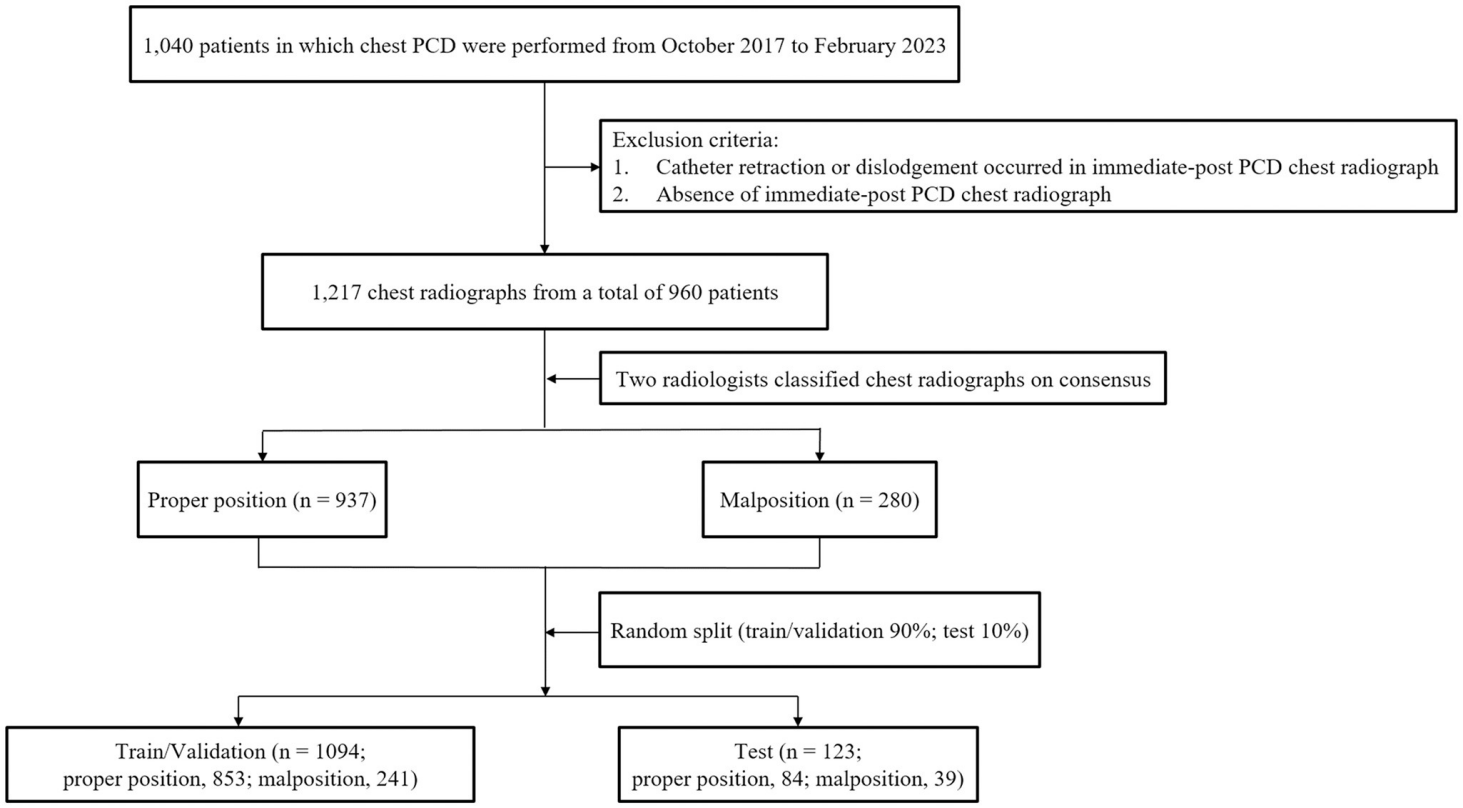
Flowchart of the case accrual process.

Plain erect posteroanterior and supine anteroposterior chest radiographs were used for the automatic detection and evaluation of the position of the catheter using deep learning in this retrospective study. The Institutional Review Board of Jeju National University Hospital approved this study (JEJUNUH 2023-04-025), and the requirement for informed consent was waived owing to the retrospective nature of the study. We initially obtained 1040 patients in which chest PCD procedures were performed from October 2017 to February 2023. We excluded follows: 1) catheter retraction or dislodgement occurred in immediate-post PCD chest radiograph (n = 66), 2) absence of immediate-post PCD chest radiograph (n = 14). Finally, we obtained 1,217 chest radiographs from a total of 960 cases. All chest radiographs were retrospectively reviewed and classified into the following two categories according to the position of the catheter by two fellowship-trained interventional radiologists with 4 and 6 years of clinical experience on consensus, to be a benchmark and serve as the reference standard for evaluation of algorithm’s performance: properly positioned (n = 937) or malpositioned (n = 280). The radiographs with no retraction nor pigtail dislodgement of the catheter compared with the immediate post-PCD radiograph were classified as properly positioned. In contrast, the radiographs with retraction or pigtail dislodgement of the catheter compared with the immediate post-PCD radiograph were classified as malpositioned. And bounding boxes were drawn at the catheter tips using Image J software (version 1.8.0, National Institutes of Health, Bethesda, MD, United States). The radiographs were randomly allocated to the training/validation (1,094 radiographs), and test (123 radiographs) datasets and 5-fold cross-validation was employed. The training/validation dataset comprised 853 radiographs with properly positioned catheters and 241 radiographs with malpositioned catheters. The test dataset comprised 84 radiographs with properly positioned catheters and 39 radiographs with malpositioned catheters. The test dataset was used to evaluate the performance of the artificial intelligence (AI) model in detecting catheter tip and classifying positions of the catheter during chest PCD on the radiographs. Fig 1 illustrates the case accrual process.

An additional analysis of the 1,217 chest radiographs was performed to investigate the association between the position and function of the catheter during chest PCD. The function of the catheter during PCD was evaluated by the drainage volume, the necessity for catheter exchange or reposition, and the improvement of pleural lesion on chest radiographs. All chest radiographs were stored in de-identified digital imaging and communication in medicine (DICOM) format using PACS.

### Image preprocessing

We applied contrast-limited adaptive histogram equalization (CLAHE) technique using the built-in function in Python (version 3.10.12) to the original DICOM images. The parameters of the augmentation process were as follows: albumentations, Blur (p = 0.01, blur_limit = (3, 7)); MedianBlur (p = 0.01, blur_limit = (3, 7)); ToGray (p = 0.01), CLAHE (p = 0.01, clip_limit = (1, 4.0); tile_grid_size = (8, 8). In standard chest radiographs, both lungs and other adjacent organ regions are present, which may obstruct catheter detection. Consequently, we utilized a center cropping method on the original DICOM images, excising about 100 pixels from the periphery. These cropped DICOM images were resized to dimensions of 2048 x 2048 pixels. To guarantee robust performance despite limited data size, we applied augmentation techniques including Blur, ToGray, and Scale.

### Deep learning architecture and training details

The PyTorch framework (Pytorch 1.13.0) was used for network training. We employed the YOLOv5 detection model, known for its high accuracy and rapid inference speed, for catheter detection. YOLOv5 utilizes CSPDarknet53 as its backbone, incorporating an optimized version of SPP (Spatial Pyramid Pooling), namely SPPF, and CSP-PAN as the neck, along with YOLOv3’s head. Available in sizes ranging from nano to extra-large, we utilized the small version (Yolov5s), which demonstrated the best performance-to-memory ratio. Subsequently, the model was fine-tuned for our specific task. Network training was performed for 25 epochs using the Adam optimizer. For loss computation, we combined three types of losses: Classes (Binary Cross Entropy loss), Objectness (Binary Cross Entropy loss), and Location (Complete IoU loss). Given the individual variability in catheter features, we applied a multi-scale training approach using images of various sizes. The learning rate and weight decay factor were set to 0.00117 and 0.00049, respectively, and to ensure stable training, we utilized a Warmup and Cosine LR scheduler. All hyperparameters were determined through Hyperparameter evolution, an optimization method using a Genetic Algorithm. Before training, we assigned a negative label (0) to "properly positioned" and a positive label (1) to "malpositioned". All training and testing phases were conducted using an RTX 3080 GPU. After multiple experiments, the model with the lowest mean Average Precision (mAP) was selected from the validation dataset to perform inferences on the test dataset. And for the detected catheter, the results are initially output as probability scores, but ultimately, a binary treatment is applied using a threshold of 0.5.

### Statistical analysis

The performance of the model in assessing the position of the catheter on the chest radiographs was described per radiographs using accuracy, sensitivity, specificity, positive predictive values (PPV), and negative predictive values (NPV) for the test dataset. In per radiographs, they were labeled as positive if all instances were positive, and the rest were labeled as negative. In per instances, the performance of the model was described using mAP50, precision and recall. Regarding the association between the position and function of chest PCD, the sensitivity and specificity were evaluated. Continuous variables are expressed as mean ± standard deviation. The Chi-square test was used to examine the independence of the two categorical variables. All statistical analyses were performed using SPSS version 22 (IBM Corp, Armonk, NY, USA) and python (version 3.10.12).

## Results

We acquired 1,217 chest radiographs from 960 patients who underwent chest PCD insertion (793 men, 167 women; mean age: 71.6 ± 0.41 years). The demographics of patients was summarized in Table 1.

**Table 1:** Demographics of patients who performed chest PCD insertion.

Age	71.6 ± 0.41 years
Sex	793 men; 167 women
Reason for PCD (%)	
Pleural effusion	880 (91.7)
Empyema	36 (3.8)
Hemothorax	15 (1.6)
Pneumothorax	13 (1.4)
Lung abscess	16 (1.7)

In per radiographs, the accuracy of the selected AI model for the test data comprising 123 radiographs was 0.88, indicating that 108 of the 123 radiographs were evaluated correctly. And the sensitivity, sensitivity, PPV, and NPV were 0.86 (95% CI: 0.78, 0.93), 0.92 (95% CI: 0.84, 1.00), 0.75 (95% CI: 0.63, 0.87) and 0.96 (95% CI: 0.92, 1.00) respectively. In per instances, the mAP50 of the selected AI model for the test data comprising 132 instances was 0.86. The precision and recall were 0.90 and 0.79 respectively. The performance results of the selected AI models were summarized in Tables 2 and 3.

**Table 2:** Outcomes of the performance test for the classification of properly positioned and malpositioned catheters per radiographs (n = 123).

	Outcomes
Accuracy	0.88
Sensitivity	0.86 [0.78–0.93]
Specificity	0.92 [0.84–1.00]
PPV	0.75 [0.63–0.87]
NPV	0.96 [0.92–1.00]

Note—Values in parentheses are the 95% confidence intervals.

*15 out of 123 images were misclassified. Abbreviation: PPV, positive predictive value; NPV, negative predictive value

**Table 3:** Outcomes of the performance test for the classification of properly positioned and malpositioned catheters per instances (n = 132).

Outcomes	Labels		
	Malpositioned	Properly positioned	All
mAP50	0.80	0.93	0.86
Precision	0.90	0.89	0.90
Recall	0.68	0.90	0.79

Note—mAP50 = mean Average Precision 50.

**Fig 2:**
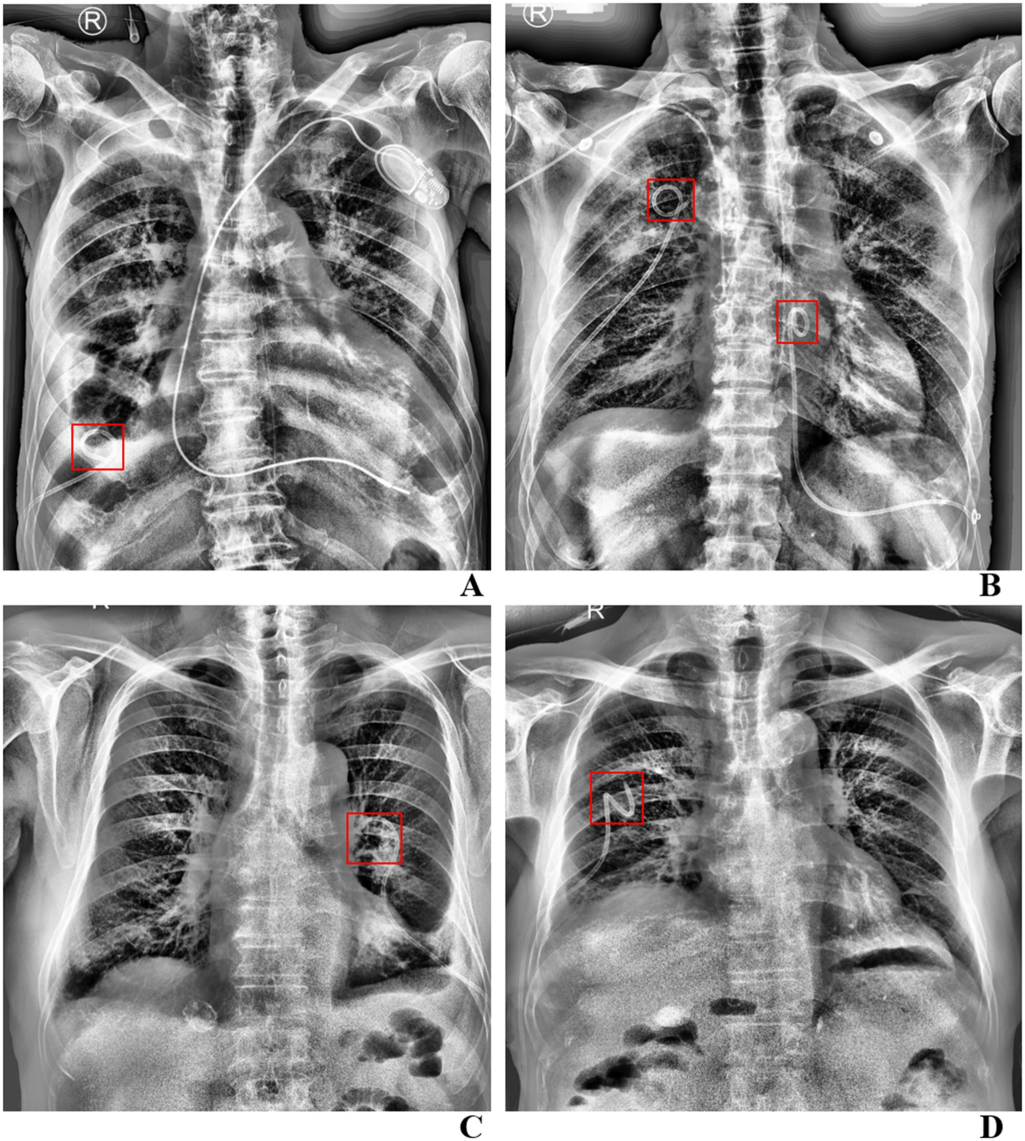
Dataset was split into 80% for training (589 of 715), 10% for validation (66 of 715), 10% for test (60 of 715).

The inference images for the test data are presented in Fig 2. The tips of the catheter were effectively detected and evaluated, regardless of the number of catheter or the presence of other medical devices such as pacemakers, PICCs, and Levin-tubes.

A. An 84-year-old male patient was performed chest PCD due to pleural effusion. The chest radiograph indicates the presence of both a chest PCD and a pacemaker. The AI prediction was properly positioned (label was properly positioned). B. A 79-year-old male patient was performed chest PCD due to bilateral pleural effusion. The chest radiograph indicates the presence of two chest PCDs, a PICC line and a Levin tube. The AI prediction was properly positioned (label was properly positioned). C. An 84-year-old female patient was performed chest PCD due to pleural effusion. The AI prediction was malpositioned (label was malpositioned). D. An 81-year-old male patient was performed chest PCD due to pleural effusion. The AI prediction was properly positioned (label was malpositioned). PCD, percutaneous catheter drainage.

Regarding the association between the position and function of chest PCD, among the 937 chest radiographs labeled as “properly positioned,” 872 cases (93%) demonstrated good function, whereas among the 280 chest radiographs labeled as “malpositioned”, 266 cases (95%) demonstrated malfunction (*p* < 0.001). Sensitivity and specificity were 0.93 (95% CI: 0.91, 0.95) and 0.95 (95% CI: 0.92, 0.98), respectively. The results were summarized in Table 4.

**Table 4:** Relationship between the position of the catheter and function on chest radiographs.

		PCD function		Total	*P* value
		Good function	Malfunction		
PCD position	Properly positioned	872	65	937	*P* < 0.001
	Malpositioned	14	266	280	
Total		886	331	1,217	
Sensitivity		0.93 [0.91–0.95]			
Specificity		0.95 [0.92–0.98]			

Note—Values in parentheses are the 95% confidence intervals. Abbreviation: PCD, percutaneous catheter drainage

## Discussion

This study aimed to automatically evaluate the position of the catheter during chest PCD on chest radiographs using deep learning. Radiologists often evaluate the position of the catheter during chest PCD on radiographs, as malposition of the catheter can result in inadequate drainage of pleural effusions, empyema, hemothorax, pneumothorax or lung abscess, leading to worsened symptoms such as dyspnea or fever. An automated solution to evaluate the catheter position can be used for the early identification of malposition. Moreover, it can assist radiologists and clinicians in repositioning catheters during chest PCD.

Most previous studies have used computerized methods to assess the position or presence of tubes, such as PICC, CVC, and ETT on radiographs [10–18]. However, the tubes have the ideal position in these studies. For instance, the optimal position of the PICC or CVC tip is between the right atrium and the superior vena cava junction [19]. In contrast, a predetermined ideal position was not used in our study during the assessment. Therefore, we defined the ideal position of the catheter as the initial position determined by the interventional radiologist and evaluated the positional changes in subsequent radiographs. Good function was observed in 93% of the patients with proper position, whereas malfunction was observed in 95% of the patients with malposition. These findings provide sufficient justification for the ideal position of the catheter during chest PCD used in this study.

In per radiographs, the accuracy of the selected AI model for the test data was 0.88. And the sensitivity, specificity, PPV, and NPV were 0.86, 0.92, 0.75 and 0.96 respectively, indicating an acceptable diagnostic performance compared with that of other studies [11, 14, 20]. Singh et al. [11] classified critical and noncritical feeding tube placement on radiographs and reported that the AUC, specificity, and sensitivity of the pretrained model. In their study, the pretrained AUC was 0.87 (95% CI: 0.80, 0.94), sensitivity was 0.88 (95% CI: 0.76, 0.95), and specificity was 0.76 (95% CI: 0.62, 0.87), respectively. Lakhani et al. [14] classified ETT positions on chest radiographs and reported that the sensitivity and specificity were 93.9% (95% CI: 90.0, 96.7) and 97.7% (95% CI: 96.9, 98.3) for detecting ETT-carina distance less than 1 cm. Thus, compared with that of previous studies [11, 14, 20], the diagnostic performance of the selected AI model in the present study was considered acceptable.

Weak labels, where all images were classified as either ’properly positioned’ or ’malpositioned, require greater training data, whereas strong labels using bounding box labeling require fewer training image datasets [14]. We used strong labels, which is an additional process to focus on the tip of the catheter by radiologists or medical imaging experts. Although our study had a relatively small sample size, by utilizing strong labels through the drawing of bounding boxes, we were able to enhance the performance of the AI model even in situations where datasets required for training were limited. The strong labels allowed us to mitigate the drawbacks of a small sample size in our study.

There are multiple radiographs obtained from a single patient. However, considering the variations in posture and patient conditions, it is challenging to regard them as identical radiographs. Additionally, the radiographs were randomly allocated within the dataset. Therefore, we concluded that the likelihood of overfitting is not high.

The majority of patients included in this study (91.7%) underwent chest PCD insertion to drain pleural effusion, suggesting that these patients could have chance to share similar clinical and imaging characteristics. However, there were variations in age, sex, characteristics of effusion, etiology of effusion, and the presence of patients with additional medical devices such as pacemaker, PICC line, and Levin-tube, aside from Chest PCD. Additionally, the number of Chest PCD instances also differed. Therefore, the clinical and imaging characteristics of the radiographs used in this study are diverse, and the test dataset can be considered as an unseen dataset.

The AI model proposed in the present study automatically detected and evaluated the position of the catheter during chest PCD. Previous studies [11, 14, 20] have also applied AI models to evaluate the catheter position; however, evaluating the position of a catheter does not necessarily indicate or predict the function. In our study, the 1,217 chest radiographs were analyzed to investigate the association between the position and function of the catheter during chest PCD. Among the 937 chest radiographs labeled as “properly positioned,” 872 (93%) demonstrated good function, whereas among the 280 chest radiographs labeled as “malpositioned,” 266 (95%) demonstrated malfunction with statistical significance with acceptable sensitivity and specificity, 93% and 95%, respectively. A significant correlation was observed between the position and function of the chest PCD, suggesting that classifying the position of the catheter during chest PCD could be valuable in clinical practice and that evaluating the PCD position could act as a factor in predicting PCD function. However, clinicians should verify the drainage pattern in cases where the AI model predicts the position of the catheter, as it is crucial to ensure the accurate diagnosis and treatment of patients.

This study has several limitations. First, although the AI model was trained using strong labels, this was a single-center study with a relatively small sample size, and there may be a risk of overfitting the AI model. Second, the sensitivity was relatively lower than the other parameters. The observed imbalance in the dataset, with a larger number of images labeled as properly positioned compared with those labeled as malpositioned, is a possible contributing factor. In the future, a larger balanced dataset must be obtained from multiple centers to train the model.

In conclusion, the AI model for the automatic detection and evaluation of the catheter position on chest radiographs demonstrated an acceptable diagnostic performance and could assist radiologists and clinicians in the early detection of malposition of the catheter during chest PCD and malfunction.
