# Correction: Detection and position evaluation of chest percutaneous drainage catheter on chest radiographs using deep learning

**DOI:** 10.1371/journal.pone.0323951

**Published:** 2025-05-27

**Authors:** Duk Ju Kim, In Chul Nam, Doo Ri Kim, Jeong Jae Kim, Im-kyung Hwang, Jeong Sub Lee, Sung Eun Park, Hyeonwoo Kim

In Table 1, there is an error in the demographics of patients’ age. The correct patients’ age should be ±. Please see the correct Table 1 here.

**Table 1:** Demographics of patients who performed chest PCD insertion.

Age	71.0 ± 15.1 years
Sex	793 men; 167 women
Reason for PCD (%)	
Pleural effusion	880 (91.7)
Empyema	36 (3.8)
Hemothorax	15 (1.6)
Pneumothorax	13 (1.4)
Lung abscess	16 (1.7)

The caption for Fig 2 is incorrect. Please see the correct Fig 2 caption here.

**Fig 2:**
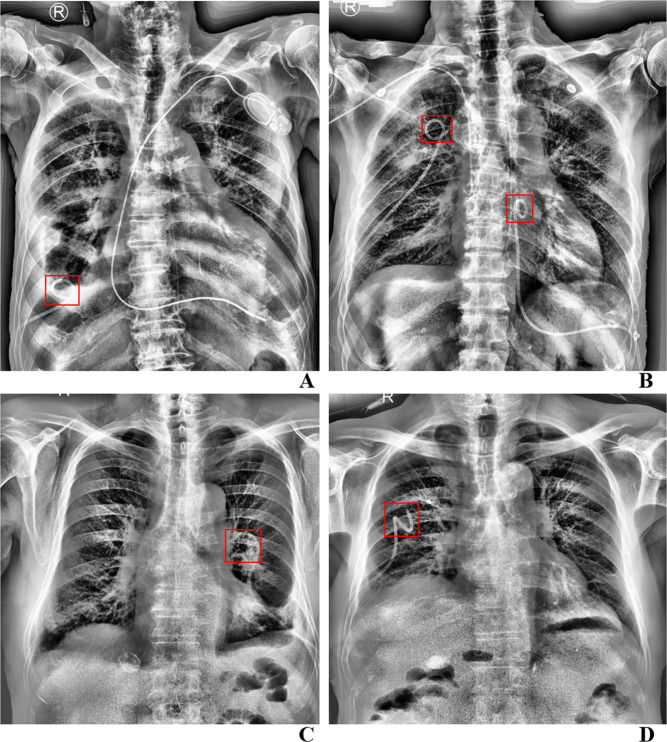
The examples of inference images for the test data.

## References

[1] Kim DJ, Nam IC, Kim DR, Kim JJ, Hwang I-K, Lee JS, et al. Detection and position evaluation of chest percutaneous drainage catheter on chest radiographs using deep learning. PLoS One. 2024;19(8):e0305859. doi: 10.1371/journal.pone.0305859 39133733 PMC11318879

